# MOSs-String-Triggered Silicon-Controlled Rectifier (MTSCR) ESD Protection Device for 1.8 V Application

**DOI:** 10.3390/mi14030632

**Published:** 2023-03-10

**Authors:** Ruibo Chen, Hao Wei, Hongxia Liu, Fei Hou, Qi Xiang, Feibo Du, Cong Yan, Tianzhi Gao, Zhiwei Liu

**Affiliations:** 1 Key Laboratory for Wide-Band Gap Semiconductor Materials and Devices of Education, School of Microelectronics, Xidian University, Xi’an 710071, China; 2 State Key Laboratory of Electronic Thin Films and Integrated Devices, University of Electronic Science and Technology of China, Chengdu 610056, China

**Keywords:** ESD protection, silicon-controlled rectifier (SCR), NMOS, trigger voltage

## Abstract

In this work, a new low voltage-triggered silicon-controlled rectifier named MTSCR is realized in a 65 nm CMOS process for low voltage-integrated circuits electrostatic discharge (ESD) protections. The MTSCR incorporates an external NMOSs-string, which drives the internal NMOS (INMOS) of MTSCR to turn on, and then the INMOS drive SCR structure to turn on. Compared with the existing low trigger voltage (V_t1_) ESD component named diodes-string-triggered SCR (DTSCR), the MTSCR can realize the same low V_t1_ characteristic but less area penalty of ~44.3% reduction. The results of the transmission line pulsing (TLP) measurement shows that the MTSCR possesses above 2.42 V holding voltage (V_h_) and a low V_t1_ of ~5.03 V, making it very suitable for the ESD protections for 1.8 V input/output (I/O) ports in CMOS technologies.

## 1. Introduction

With the scaling down of CMOS processes, the challenges of the electrostatic discharge (ESD) protection for integrated circuits (IC) become more severe [1]. The gate-grounded NMOS (GGNMOS), which was previously one of the most widely used ESD protection devices in CMOS processes, is invalid in advanced CMOS technologies due to its inadequate robustness [2,3,4]. Therefore, the researchers began to turn their attentions to the lateral silicon-controlled rectifier (LSCR) thanks to its higher level of robustness. However, the conventional LSCR shows a deep snap-back in its I-V characteristic, which violates the ESD design window of most IC processes. Improved designs have been constantly proposed to enable SCR structure to be applied in practical projects [5,6], such as the modified lateral SCR (MLSCR) [7,8] and the diodes-string-triggered SCR (DTSCR) [9,10,11,12,13,14]. As such, the DTSCR is particularly well-suited for lower voltage domain with benefit from its lower and adjustable trigger voltage [9]. Moreover, the improved structures based on DTSCR have been continually proposed. For example, in [15], a novel device called thermal-stable DTSCR (TSDTSCR) is proposed to offer an improved ESD protection stability at elevated temperatures, which is realized by optimizing the 3D layout of the DTSCR. In another work, researchers embedded current gain amplifier modules into DTSCR and achieved faster turn-on speed and superior I-V properties [16].

Nevertheless, the DTSCR structures are used to protect the circuits that operate below 1.8 V. For 2.5 V or above circuits ESD protections, the DTSCR structures will incorporate more than four triggering diodes for voltage clamp, and will have enlarged leakage current due to the enhanced Darlington effects. In addition, the area consumption will also increase with more numbers of the triggering diode. In recent years, a SCR structure called directly connected SCR (DCSCR) has been reported [17], which is independent of external diodes-string to trigger. However, the DCSCR is aimed at the ESD protection of core circuit and the rail-based ESD protection scheme. For the local-based ESD protection scheme of input/output (I/O) circuit, DCSCR has the issues of mis-trig risk and high leakage. At this point, low voltage-triggered SCR (LVTSCR) has exhibited sufficient area-efficiency for low voltage ESD protections in deep sub-micron technologies, but it will be invalid in advanced processes due to its intolerable I-V characteristics [18,19,20,21]. Many new structures based on LVTSCR have been proposed with higher holding voltage [22,23], and there are also studies focusing on improving the on-resistance of LVTSCR structure [24]. However, the high trigger voltage of LVTSCR structure is still a critical issue for its application in advanced processes.

This paper proposed a MOS-string-triggered SCR (MTSCR) as a more appropriate ESD protection solution for the 1.8 V circuits, with less area consumption and immunity on the Darlington effect compared to the DTSCR structures. 

## 2. Methods

**Figure 1:**
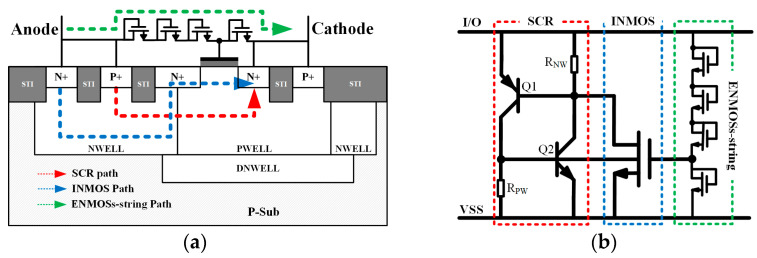
The proposed MTSCR device: (**a**) cross-sectional view and (**b**) equivalent circuit.

Figure 1a shows the cross-sectional view of MTSCR, and its equivalent circuit is presented in Figure 1b. The MTSCR incorporates an internal NMOS (INMOS) within the PWELL, with its drain spanning the boundary between NWELL and PWELL. In addition, an external NMOS (ENMOS) string is paralleled to the SCR structure. The gate and drain of ENMOS are tied together. Moreover, the gate of the INMOS is connected to the drain of one ENMOS in ENMOSs-string. When an ESD event arrives, it will force the ENMOSs-string path to conduct, then the INMOS will turn on as the voltage on its gate exceeds the threshold voltage. Meanwhile, the emitter-base junctions of Q1 and Q2 can be charged, and eventually, the SCR path is triggered. It should be noted that the INMOS acts as a similar current-trigger assistance effect as the diodes-string of the DTSCR, whereas the ENMOSs-string merely dominates the turn on of the INMOS rather than the parasitic bipolar transistors Q1 and Q2 of the inherent SCR. Therefore, the ENMOSs-string can be designed with a much smaller equivalent width to realize a larger resistance, which will prompt more current to the INMOS current-trigger path.

## 3. Results and Discussion

**Figure 2:**
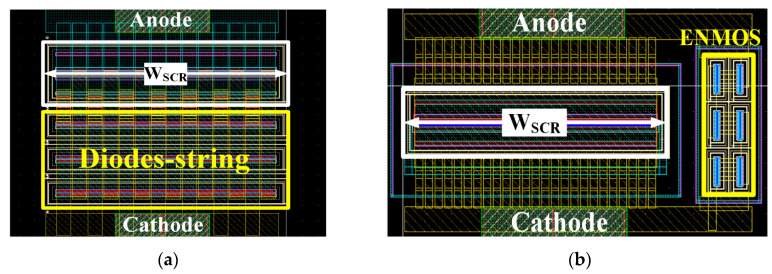
Layouts of (**a**) the conventional DTSCR and (**b**) the proposed MTSCR device.

In this paper, the proposed MTSCR with different numbers and different widths of ENMOS (W_N_) transistors are realized and evaluated in a 65 nm CMOS logic technology. All ENMOS transistors of the ENMOSs-string and INMOS introduce the 1.1 V standard specification with the channel length L of 0.28 µm, and their V_th_ are ~0.345 V. With the consideration of 1.8 V circuits ESD protection applications, the MTSCR with six ENMOS transistors and the conventional DTSCR with three trigger-diodes are compared. Their layouts are as shown in Figure 2a,b, where the inherent SCRs of the two types devices have the same area of ~770 μm^2^ and the same width (W_SCR_) of 55 μm. In order to realize acceptable trigger characteristics of the DTSCR, the diodes-string is always kept the same to the equivalent width of the inherent SCR. While the ENMOSs-string of the MTSCR is designed with a much smaller width of 5 μm, it will not worsen the trigger characteristics. Eventually, the total area consumption of MTSCR is 1040 μm^2^, which is ~50% lower than the 2000 μm^2^ area consumption of DTSCR. The innovativeness of MTSCR is that it incorporates an ENMOSs-string on the basis of the LVTSCR, thus eliminating the Darlington effect that was brought by the diodes-string in the DTSCR and also reducing area consumption. The quasi-static I-V characteristics of the proposed devices are measured using Hanwa TED-T5000 transmission line pulsing (TLP) tester with 10 ns rise time and 100 ns pulse width, and their CDM ESD characteristic were evaluated by ESDEMC MODEL ES620 Very Fast TLP (VF-TLP) tester with 200 ps rise time and 5 ns pulse width.

**Figure 3:**
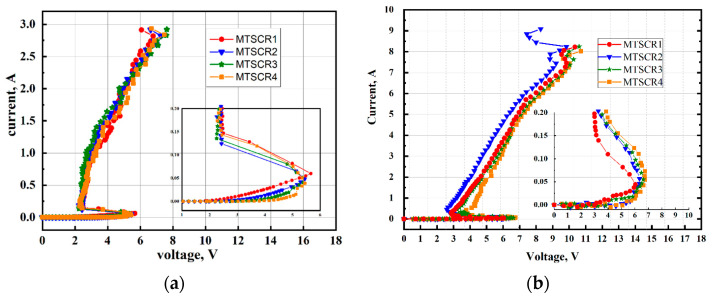
(**a**) TLP and (**b**) VF-TLP test results of MTSCRs with N = 3, 4, 5, and 6. The rise time of the TLP and VFTLP pulse are 10 ns and 200 ps, respectively.

Four different MTSCRs with varying numbers (N = 3, 4, 5, 6) of ENMOSs are labeled as MTSCR1, MTSCR2, MTSCR3, and MTSCR4. The TLP I-V characteristics of these devices are shown in Figure 3a, and the extracted parameters are compared in Table 1. It can be observed from Figure 3a that all devices exhibit two stages during their trigger processes. For MTSCR1, the first stage commences at about 1.92 V (Von), which is caused by the INMOS being turned on by ENMOSs-string. Then, the second stage occurs at about 5.65 V, where the SCR path is triggered. It can also be observed that as N increases from 3 to 6, the Von is increased from 2.8 V to 4.05 V, while the V_tl_ is decreased from 5.65 V to 5.47 V. Indeed, the ENMOSs-string only dominates the conduction of INMOS’s channel by controlling the gate voltage of the INMOS. Since the INMOS turned on, the current will be discharge by the ENMOS path and INMOS path, while the trigger voltage of the MTSCR is actually determined by the turn-on of the SCR path, which is dominated by its parasitic transistors Q1 and Q2. As the number of the ENMOS (N) increased, more current will be prompted from the ENMOS path to the INMOS path. As discussed in the Section 2 Methods, the real current trigger path of MTSCR is the INMOS path, thus the increased N will urge more current flow to the INMOS path, and consequently both the total trigger current and trigger voltage of MTSCR are lowered. It should be noted that the V_h_, and I_t2_ of MTSCRs remain consistent. Hence, it is anticipated that MTSCR can suit various ESD design windows by modifying the number of ENMOSs accordingly.

Figure 3b presents the VF-TLP test results of the four MTSCRs. Parameters are extracted into Table 1. From Table 1, an interesting phenomenon can be observed: the V_tl_ of TLP and VFTLP show the opposite trend. This is because the rise time and duration of VF-TLP pulse are far shorter than that of TLP [25]; therefore, when the time reaches the 70% of one VF-TLP pulse, the device has not reached steady state and the voltage is still on the process of lowering, that is to say, the turn-on time can affect the V_tl_ of the device under VF-TLP test. If the number of ENMOS is increased, lower voltage will be coupled to the gate of the INMOS, and consequently the INMOS path will be turned on later, showing an increased trigger voltage under VF-TLP test.

**Figure 4:**
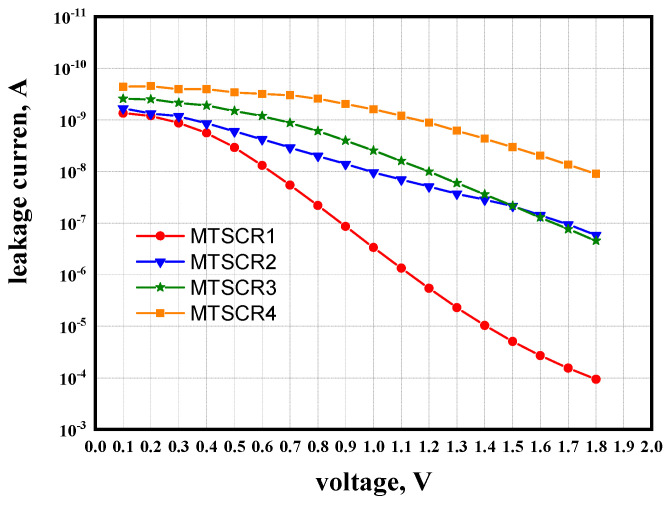
DC sweep of MTSCR1, MTSCR2, MTSCR3, and MTSCR4 at 25 °C.

Figure 4 shows the leakage of MTSCRs at 25 °C, which indicates that the leakage decreases with the increase in the number of ENMOS. For 1.8 V applications in 65 nm CMOS process, the MTSCR4 with the leakage of ~10 nA can satisfy the ESD design window, which is between 1.98 V and 8.1 V. However, it is worth noting that the leakages of the MTSCRs are dominant by the total V_th_ of the ENMOSs-string, and consequently incorporating higher V_th_ ENMOS transistors instead of 1.1 V standard specification might optimize the leakage characteristic without increasing the ENMOS’s number. 

**Figure 5:**
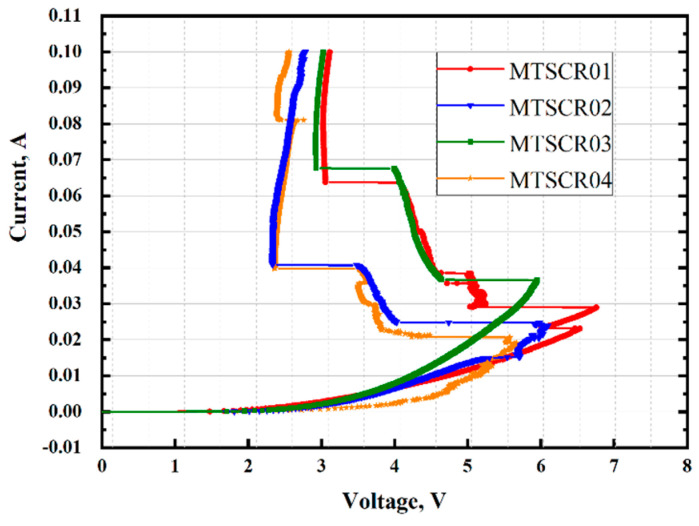
DC sweep of MTSCR1, MTSCR2, MTSCR3, and MTSCR4 at 150 °C.

Static latch-up evaluation has been checked with DC I-V characteristic of the proposed devices at 150 °C, which is shown in Figure 5. The current was restricted to below 0.1 A to protect the tested devices. The results shows that the V_h_ of all devices exceed 1.98 V. Therefore, the proposed MTSCR can provide steady ESD protection for 1.8 V of supply voltage circuits without latch-up risk.

**Figure 6:**
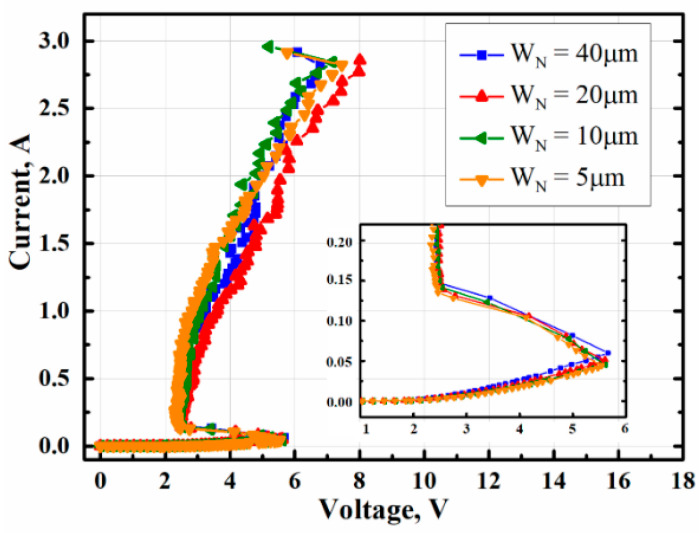
TLP test results of MTSCRs with different widths of the ENMOS transistors (W_N_).

The MTSCR devices with different width of ENMOSs W_N_ are evaluated by TLP measurement as shown in Figure 6. It is clearly observed that the I_t2_ of the devices has no reduction with the W_N_ decreased. This is because the ENMOSs-string of the MTSCR dominates the gate voltage bias of the INMOS. Since the INMOS turned on, the inherent SCR of the MTSCR will be triggered by the INMOS current assistance, and then most of the ESD current will shunt to the inherent SCR path.

**Figure 7:**
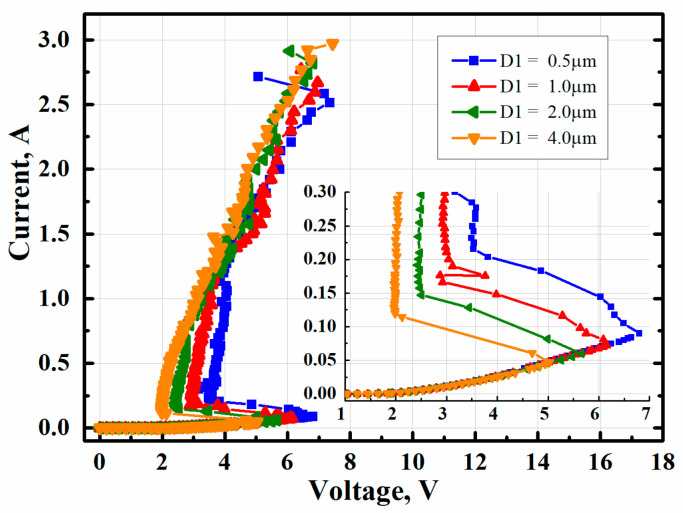
TLP test results of MTSCRs with four different D1.

In modern technology, the epitaxy is getting thinner and thinner, and the sheet resistance of WELL is also increasing with it. Thus, the impact of D1 (shown in Figure 1a) on well resistance will be significant. Figure 7 presents the TLP test results of MTSCRs with various D1. As D1 was increased from 0.5 μm to 4.0 μm, the V_t1_ decreased from 6.92 V to 5.03 V. The trigger current also decreased from 892 mA to 467 mA, while the I_t2_ increased from 2.72 A to 2.97 A. This phenomenon is related to the current distribution among the current within the MTSCR. The increased D1 means larger equivalent resistance of INMOS path and N+/NWELL/PWELL/P+ diode path, resulting in more current flow to the SCR path, which leads to the decreasing V_tl_ and trigger current, and increasing I_t2_.

**Figure 8:**
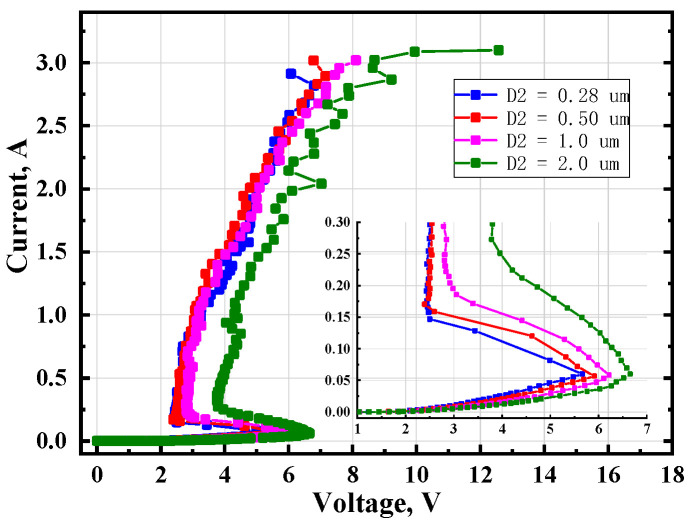
TLP test results of MTSCRs with four different D2.

For 2.5 V circuits ESD protections, the holding voltage of the MTSCR can be further improved by adjusting the dimension of D2 as shown in Figure 1a. As it is shown in Figure 8, when increasing D2, the well resistance in SCR path will be enlarged, and consequently both the V_h_ and V_t1_ will be increased.

Table 2 summarizes the ESD characteristics of the proposed MTSCR and the structures in [24,26,27]. The DCSCR possesses a very high It2, but the low trigger voltage determines that it cannot satisfy the ESD design window of 1.8 V circuit. As for the improved LVTSCR, although it has high holding voltage, its trigger voltage is exorbitant. Both the DTSCR and the MTSCR can be adjusted to fit the 1.8/2.5 V circuits, but MTSCR consumes less area and possesses much higher It2 than the DTSCR. Thus, the MTSCR has advantages for the ESD protection of 1.8/2.5 V circuit.

## 4. Conclusions

In this paper, a novel and improved low-triggered ESD protection structure called MTSCR has been designed and fabricated in a 65 nm CMOS process. When compared with the DTSCR, the MTSCR is able to achieve a low trigger voltage as well as ~44.3% reduction in area consumption. The MTSCR also possesses tunable trigger voltage and low leakage that is nA-level, which makes it more advantageous in the ESD protection of 1.8 V voltage domain. Moreover, the MTSCR can suit the ESD design window of 2.5 V voltage domain by adjusting D1 and the number of ENMOS transistors.

## Figures and Tables

**Table 1:** TLP and VF-TLP test results of MTSCRs.

Device Name	Number of NMOS (N)	TLP Measurement Results				VF-TLP Measurement Results			
		V_on_ (V)	V_t1_ (V)	V_h_ (V)	I_t2_ (A)	V_on_ (V)	V_t1_ (V)	V_h_ (V)	I_t2_ (A)
MTSCR1	3	1.92	5.65	2.42	2.72	3.14	6.03	2.85	7.86
MTSCR2	4	2.80	5.51	2.50	2.71	4.25	6.37	2.60	8.09
MTSCR3	5	3.44	5.42	2.21	2.71	4.99	6.62	3.23	8.18
MTSCR4	6	4.05	5.47	2.35	2.73	5.39	6.75	3.68	7.43

**Table 2:** Comparison among the DTSCR, DCSCR, improved LVTSCR, and the proposed MTSCR.

SCR Category	Technology Process	Trigger Voltage (V)	Area (μm^2^)	FOM (mA/um^2^)	Applicable Voltage Domain
DTSCR with 3 diodes [26]	0.18 μm CMOS	2.6	2527	1.58	1.8 V
DCSCR [27]	0.18 μm CMOS	1.6	1551	3.67	0.8–1.2 V
Improved LVTSCR [24]	0.18 μm BCD	9.14	2952	2.46	5 V
This work	65 nm CMOS	5.47	1040	2.62	1.8–2.5 V

## Data Availability

Not applicable.

## References

[1] Duvvury C. ESD qualification changes for 45 nm and beyond IEEE Int. Proceedings of the 2018 Electron Devices Meeting.

[2] Du F.-B., Liou J.J. (2019). An enhanced gate-grounded NMOSFET for robust ESD applications. IEEE Electron Device Lett..

[3] Li J., Halbach R. Analysis of failure mechanism on Gate-Silicided and Gate-Non-Silicided, Drain/Source Silicide-blocked ESD NMOSFETs in a 65 nm Bulk CMOS technology int. Proceedings of the 2006 13th International Symposium on the Physical and Failure Analysis of Integrated Circuits.

[4] Kim C.S., Park H.B., Kim B.G., Kang D.G., Lee M.G., Lee S.W., Jeon C.H., Kim W.G., Yoo Y.J., Yoon H.S. A novel NMOS transistor for high performance ESD protection devices in 0.18/spl mu/m CMOS technology utilizing salicide process. Proceedings of the Electrical Overstress/Electrostatic Discharge Symposium Proceedings 2000 (IEEE Cat. No. 00TH8476).

[5] Ker M.-D., Hsu K.C. (2005). Overview of on-chip electronstatic discharge protection design with SCR-base devices in CMOS integrated circuits. IEEE Trans. Device Mater. Rel..

[6] Sarro J., Rosenbaum E. Study of design factors affecting turn-on time of silicon controlled rectifiers (SCRS) in 90 and 65 nm bulk CMOS technologies. Proceedings of the 2006 IEEE International Reliability Physics Symposium Proceedings.

[7] Du F., Hou F., Song W., Chen R., Liu J., Liu Z., Liou J.J. (2019). An enhanced MLSCR structure suitable for ESD protection in advanced epitaxial CMOS technology. IEEE Trans. Electron Devices..

[8] Lu T.C., Wang M.T., Shone F. Design strategy of MLSCR devices for sub-micron CMOS technology. Proceedings of the Technical Papers. International Symposium on VLSI Technology, Systems, and Applications.

[9] Mergens M. (2005). Speed optimized diode-triggered SCR (DTSCR) for RF ESD protection of ultra-sensitive IC nodes in advanced technologies. IEEE Trans. Device Mater. Rel..

[10] Gauthier R., Li J. Investigation of voltage overshoots in diode triggered silicon controlled rectifiers (DTSCRs) under very fast transmission line pulsing (VfTLP). Proceedings of the 2009 31st EOS/ESD Symposium.

[11] Chen W.-Y., Ker M.-D. (2012). Diode-triggered silicon controlled rectifier with reduced voltage overshoot for CDM ESD protection. IEEE Trans. Device Mater. Rel..

[12] Miao M. (2012). Minimizing multiple triggering effect in diode triggered silicon-controlled rectifiers for ESD protection applications. IEEE Electron Device Lett..

[13] Ker M.-D., Wu W.-L. (2006). ESD-protection design with extra low-leakage-current diode string for RF circuits in SiGe BiCMOS process. IEEE Trans. Device Mater. Rel..

[14] Liu J., Zhiwei L. (2017). A Diode-Triggered Silicon-Controlled Rectifier with Small Diode Width for Electrostatic Discharge Applications.

[15] Hou F., Liu J., Liu Z., Huang W., Gong T., Yu B., Liou J.J. (2019). New Diode-Triggered Silicon-Controlled Rectifier for Robust Electrostatic Discharge Protection at High Temperatures. IEEE Trans. Electron Devices.

[16] Du F., Song W. (2020). Augmented DTSCR With Fast Turn-On Speed for Nanoscale ESD Protection Applications. IEEE Trans. Electron Devices.

[17] Sun R.C., Wang Z., Klebanov M., Liang W., Liou J., Liu D.G. (2015). Silicon-Controlled Rectifier for Electrostatic Discharge Protection Solutions With Minimal Snapback and Reduced Overshoot Voltage. IEEE Electron Device Lett..

[18] Di Sarro J., Rosenbaum E. Evaluation of SCR-based ESD protection devices in 90 nm and 65 nm CMOS technologies. Proceedings of the 2007 IEEE International Reliability Physics Symposium Proceedings, 45th Annual.

[19] Chatterjee A., Polgreen T. (1991). A low-voltage triggering SCR for on-chip ESD protection at output and input pads. IEEE Electron Device Lett..

[20] Shan Y., Hu B. (2009). PLDD/NHALO-assisted low-trigger SCR for high-voltage tolerant ESD protection in foundry CMOS process without extra mask. Electron Device Lett..

[21] Ker M.-D., Chang H.-H. (1996). Complementary-LVTSCR ESD protection circuit for submicron CMOS VLSI/ULSI. IEEE Trans. Electron Devices.

[22] Yang K., Liu J., Liu Z. LVTSCR with High Holding Voltage for ESD Protection in 55 nm CMOS Process. Proceedings of the 2019 8th International Symposium on Next Generation Electronics (ISNE).

[23] Huang M., Du F., Hou F., Song W., Liu J., Liu Z. Enhanced LVTSCR with High Holding Voltage in Advanced CMOS technology. Proceedings of the 2019 IEEE International Conference on Electron Devices and Solid-State Circuits (EDSSC).

[24] Do K.I., Koo Y.S. (2020). A New SCR Structure With High Holding Voltage and Low ON-Resistance for 5-V Applications. IEEE Trans. Electron Devices.

[25] Muhonen K., Grund E., Ashton R. High-Speed TLP and ESD Characterization of Ics. Proceedings of the 2021 IEEE BiCMOS and Compound Semiconductor Integrated Circuits and Technology Symposium (BCICTS).

[26] Du X., Dong S., Han Y., Huo M., Huang D. (2009). Low-leakage diode-triggered silicon controlled rectifier for electrostatic discharge protection in 0.18-μm CMOS process. J. Zhejiang Univ. Sci. A.

[27] Liang H., Ma Q., Sun J., Liu J., Gu X. (2022). A Novel DTSCR With Embedded MOS and Island Diodes for ESD Protection of High-Speed ICs. IEEE Trans. Device Mater. Reliab..

